# Exploring the association between primary care efficiency and health system characteristics across European countries: a two-stage data envelopment analysis

**DOI:** 10.1186/s12913-023-10369-y

**Published:** 2023-12-04

**Authors:** Valerie Moran, Marc Suhrcke, Ellen Nolte

**Affiliations:** 1 Socio-Economic and Environmental Health and Health Services Research Group, Department of Precision Health, Luxembourg Institute of Health, Luxembourg City, Luxembourg; 2 Socio-Economic and Environmental Health and Health Services Research Group, Living Conditions Department, Luxembourg Institute of Socio-Economic Research (LISER), Belval, Esch-sur-Alzette, Luxembourg; 3 Department of Health Services Research and Policy, London School of Hygiene and Tropical Medicine, London, UK

**Keywords:** Primary care, Technical efficiency, Performance, Data envelopment analysis, International comparisons, European countries

## Abstract

**Background:**

Primary care is widely seen as a core component of resilient and sustainable health systems, yet its efficiency is not well understood and there is a lack of evidence about how primary care efficiency is associated with health system characteristics. We examine this issue through the lens of diabetes care, which has a well-established evidence base for effective treatment and has previously been used as a tracer condition to measure health system performance.

**Methods:**

We developed a conceptual framework to guide the analysis of primary care efficiency. Using data on 18 European countries during 2010–2016 from several international databases, we applied a two-stage data envelopment analysis to estimate (i) technical efficiency of primary care and (ii) the association between efficiency and health system characteristics.

**Results:**

Countries varied widely in terms of primary care efficiency, with efficiency scores depending on the range of population characteristics adjusted for. Higher efficiency was associated with bonus payments for the prevention and management of chronic conditions, nurse-led follow-up, and a financial incentive or requirement for patients to obtain a referral to specialist care. Conversely, lower efficiency was associated with higher rates of curative care beds and financial incentives for patients to register with a primary care provider.

**Conclusions:**

Our results underline the importance of considering differences in population characteristics when comparing country performance on primary care efficiency. We highlight several policies that could enhance the efficiency of primary care. Improvements in data collection would enable more comprehensive assessments of primary care efficiency across countries, which in turn could more effectively inform policymaking.

**Supplementary Information:**

The online version contains supplementary material available at 10.1186/s12913-023-10369-y.

## Background

Efficiency is considered a key measure of health system performance [1]. Assessments of overall system efficiency might conceal important variation between different parts of the health system, and it is important to understand how the various sectors perform in order to effectively inform decision-making by mangers and policymakers [2]. Related research has tended to focus on hospitals (including different types of ownership), not least due to the availiability of data [3–5] and because the hospital setting has clear boundaries [6]. Efficiency in primary care is less well understood and the imprecise boundaries and wide range of outputs mean that efficiency is more difficult to assess [2, 7, 8]. Yet, primary care is recognised to be at the core of resilient and sustainable health systems [9], with evidence pointing to its key role in improving health outcomes, health system efficiency and health equity [10, 11]. Primary care also plays an important role in the effective prevention and integrated management of the rising burden of chronic disease globally [12]. It is against this background that assessing the performance and efficiency of primary care has become ever more important [13].

Most studies of efficiency in primary care have been conducted in European countries, with a focus on technical efficiency of primary care providers, such as general practices, primary care centres or primary care teams within countries [8, 14, 15]. Only one study [16] specifically examined primary care efficiency across [22] European countries, finding that countries varied considerably in terms of their efficiency in translating care structures into processes and care processes into quality outcomes. The study provided important insights into technical efficiency as measured by primary care structures, such as governance, financing or workforce; processes (e.g. access, continuity of care) and outcomes (quality, efficiency, equity). However, it did not explore the factors that could explain variation between countries in terms of primary care efficiency. Such an analysis would allow for inferences about the likely contribution of national-level policies to improve efficiency [2].

This paper seeks to address this important research gap by investigating the relationship between health system characteristics and primary care efficiency across European countries. Specifically, we seek to explore whether a given characteristic is associated with higher or lower primary care efficiency. We examine this issue through the lens of diabetes care, which has a well-established evidence base for effective treatment, much of which can be delivered in primary care [17]. Diabetes has been proposed as a useful tracer condition to assess health system performance [18]. As efficiency analysis is inherently a comparative exercise, focusing on a single condition can enhance the comparability of efficiency measures across countries [2] and may facilitate the identification of outputs attributable to primary care.

## Methods

### Definition of primary care

We defined primary care in line with the European Commission (2014) as “*the provision of universally accessible, integrated person-centred, comprehensive health and community services provided by a team of professionals accountable for addressing a large majority of personal health needs. These services are delivered in a sustained partnership with patients and informal caregivers, in the context of family and community, and play a central role in the overall coordination and continuity of people’s care*.” [19] (p. 18). Our focus is on technical efficiency, which refers to the ability to maximise outputs (or outcomes) given a set of limited inputs or resources or to minimise inputs to obtain a given level of outputs [2].

### Conceptual framework of primary care performance on efficiency

**Fig. 1:**
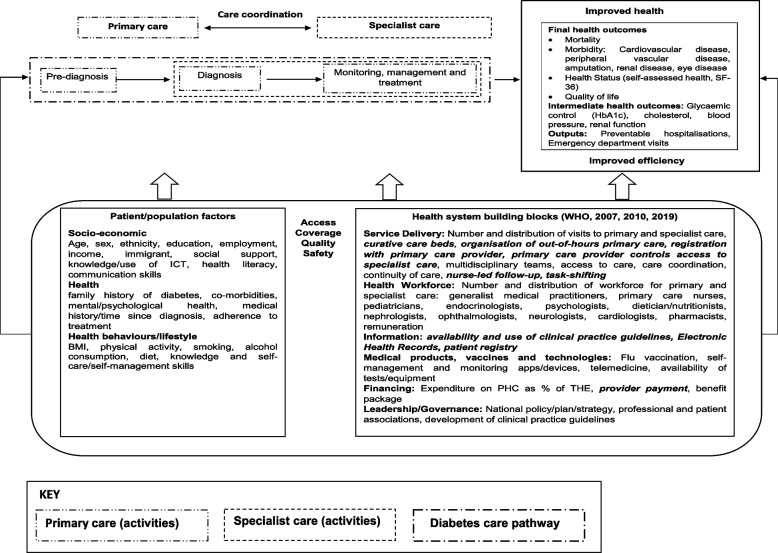
Conceptual framework. *Note:* Health system characteristics included in the analysis are highlighted in bold and italic. Source: Modified based on Brown et al. [20] and the World Health Organisation [21–23]

We developed a conceptual framework (Fig. 1). It describes a simplified pathway from pre-diagnosis to diabetes treatment and management along the primary-secondary care continuum, along with intermediate and final outcomes of diabetes care [20]. It identifies the range of factors acting at patient, population and health system levels that can impact the journey and, ultimately, the outcomes along the care continuum.

### Data

Our choice of variables was informed by the conceptual framework (Fig. 1) and data availability. We considered 18 European OECD member countries, for which relevant data on key input and output variables were available for the period 2010–2016. We merged data from ten different sources. Information on data sources, missing data and variable definition and construction is available in Supplementary file, Table S1.

#### Input and output variables

We sourced data on input and output variables from the OECD Health Statistics database [24]. The input variable was the number of generalist medical practitioners, per 1,000 population, a widely used measure of primary care resources.

We considered eight variables reflecting population characteristics as ‘uncontrollable’ inputs [25] as they are outside of the direct control of the primary care system, at least in the short term, specifically: prevalence of diabetes [26] and obesity [27]; smoking rates (population aged 15 years and over) [28]; per capita alcohol consumption [24]; levels of deprivation [29]; educational achievement [30]; per capita income [31] and the incidence of long-term (one year and over) unemployment [31]. Countries with a higher prevalence of diabetes may have higher rates of diabetes hospital admissions [32] and amputations [33]. Obesity and smoking are risk factors for diabetes hospital admission [34], and smoking is also a risk factor for lower limb amputation [35] while alcohol use has been associated with a lower risk of hospital admission [34]. Socio-demographic factors including deprivation, education, income and unemployment have also been associated with poor diabetes outcomes [20, 36–38].

Output variables were diabetes hospital admissions per 100,000 population and admissions based on diabetes lower extremity amputation, per 100,000 population, which measure utilisation and quality of primary care [33, 39]. Both variables were adjusted for age and sex. Data envelopment analysis (DEA) (detailed explanation below in the section “Two-stage data envelopment analysis”) assumes that the measurement of outputs implies ‘more is better’ [40] that is, larger numerical values correspond to greater production [41], suggesting that outputs should be maximised [42]. However, the interpretation of diabetes admission and amputation rates is that lower rates point to better quality of care. Therefore, we transform the output variables using the multiplicative inverse in order to incorporate them into the models as desirable outputs [42] that we wish to maximise.

#### Explanatory variables: Health system characteristics

Based on availability of data, we selected the following health system characteristics: number of curative care beds per 1,000 population [24], availability and use of electronic health records (EHR) by GPs [43], bonus payments for primary care providers achieving targets related to the prevention and management of chronic diseases [44], nurse-led follow-up of people with chronic conditions [44], requirement for patients to register with a primary care provider [44], requirement for patients to obtain primary care referral to specialist care [44], arrangements for out-of-hours primary care (group of physicians on a rota basis) [44–47], existence of a diabetes registry [48], existence of government-approved evidence-based national guidelines for the management of diabetes [45, 48, 49], and task-shifting from physicians to nurses in primary care [50] (for further details see Supplementary file Table S2).

We expected a higher rate of curative care beds to be associated with lower efficiency as higher rates of beds could translate into higher rates of admissions. For the remaining variables, we expected a positive association with efficiency.

### Two-stage data envelopment analysis

We used a two-stage DEA [51] to investigate the relationship between efficiency and health system characteristics. DEA is a recommended approach for measuring efficiency in the context of small sample sizes and multiple inputs and outputs [25].

First, we measured technical efficiency, using DEA, whereby efficiency is defined as the ratio of a weighted sum of outputs to a weighted sum of inputs. The DEA method chooses the set of ouput and input weights that maximise the efficiency of country *i*, subject to the constraint that the efficiency score is less than or equal to one. We assumed an output orientation, which implied that output could be increased given a specified level of input as our aim was to estimate the potential increase in primary care quality (output) that could be achieved with the available primary care resources (input). Therefore, our objective was to maximise weighted outputs conditional on weighted inputs being equal to one:

$${\mathrm{Max}}_{\mathrm u,\mathrm y}\left(\mathrm u'{\mathrm y}_0\right)$$

Subject to:

$$v'=1$$

$$u'y_i-v{'x}_i\leq0i=1,\dots.,\mathrm I$$

$$u, v\ge 0$$

where *x*_*i*_ is a vector of inputs and *y*_*i*_ is a vector in outputs for each of the *I* countries [25].

Countries with the highest ratio of output to input formed the efficiency frontier. The efficiency of countries not on the frontier was assessed relative to the most efficient countries or ‘peers’ that comprised the frontier [52]. We used the Banker, Charnes and Cooper specification i.e. variable returns to scale [53], which is recommended when outputs are expressed in ratios [54].

We adjusted efficiency scores to reflect differences in population characteristics across countries, identifying population characteristics that had a statistically significant association with efficiency and including these in the DEA models as ‘uncontrollable’ inputs’ to ensure a more meaningful comparison of countries. We first estimated a baseline model without any population characteristics; it included generalist medical practitioners as input and diabetes hospital admissions per 100,000 population and admissions for diabetes lower extremity amputation per 100,000 population as outputs. We then estimated separate DEA models for each statistically significant population characteristic to evaluate its effect on countries efficiency scores. We computed Spearman rank correlations between the efficiency estimates from the different models to assess their internal validity [3]. DEA does not account for measurement error and the efficiency frontier is an estimate of the true frontier based on our data sample. In order to adjust the efficiency estimates for sampling bias, we applied bootstrapping as proposed by Simar and Wilson [55]. We ran the bootstrap for 2,000 iterations. We computed the bias-corrected efficiency scores for each year using the *Benchmarking* package in R [56].

Second, we estimated a truncated regression with the efficiency estimates from each model as the dependent variable and health system characteristics as explanatory variables. We corrected the standard errors for sampling bias and the correlation between efficiency scores using bootstrapping with 2,000 iterations [51]. We pooled data across years to estimate the regressions. While it would be informative to model several health system characteristics variables simultaneously, we included each variable separately due to the small number of countries (n = 18). The regressions were estimated using the *truncreg* command in Stata 17 [57].

### Ethical issues/statement

Ethics approval was not required as we used secondary data that was aggregated at the country-level.

## Results

### Descriptive statistics

Table 1 shows the descriptive statistics for the input, output, population and health system characteristics variables (see also Supplementary file Figures S1-S13).

**Table 1:** Descriptive statistics of study variables

**Continuous variables, n = 106** ^**a**^				
**Variable**	**Mean**	**Standard deviation**	**Min**	**Max**
**Input variable**				
Generalist medical practitioners, per 1,000 population	0.99	0.46	0.33	2.74
**Population characteristics (uncontrollable inputs)**				
Diabetes prevalence, %	5.36	1.45	2.77	9.26
Smoking, %	27.25	4.52	18.80	37.30
Alcohol consumption, litres per capita	10.08	1.95	6.00	14.70
Obesity, % of population aged 18 years and over	21.29	2.31	17.40	27.80
Deprivation, % of population	5.92	4.30	0.50	19.80
Income per capita, US$ PPP	44746.46	16223.48	21088.60	103723.70
Percentage of total unemployed population unemployed for one year or more	36.69	12.42	16.80	61.70
Upper second level education, % of population aged 25–64 years	43.76	11.24	20.74	66.05
**Output variables**				
Diabetes hospital admissions, per 100,000 population	142.42	64.91	43.80	281.10
Admission based diabetes lower extremity amputation, per 100,000 population	6.53	3.96	2.60	24.40
**Estimated dependent variables**				
Efficiency scores, baseline model	0.58	0.21	0.17	0.90
Efficiency scores, baseline model with diabetes prevalence	0.57	0.20	0.17	0.90
Efficiency scores, baseline model with alcohol	0.71	0.17	0.31	0.92
Efficiency scores, baseline model with obesity	0.58	0.20	0.17	0.90
Efficiency scores, baseline model with smoking	0.67	0.19	0.28	0.90
Efficiency scores, baseline model with education	0.58	0.21	0.17	0.88
Efficiency scores, baseline model with income	0.61	0.21	0.17	0.91
**Health system characteristics continuous variables**				
Curative care beds, per 1,000 population	3.78	1.33	2.15	6.28
Availability and use of Electronic Health Records (EHR) by GPs	2.87	0.44	1.39	3.33
**Health system characteristics binary variables, n = 18**	**Number**	**Percentage**	**Min**	**Max**
*Bonus payment for primary care providers*				
No	11	61	0	0
Yes	7	39	1	1
*Nurse-led follow-up of patients with chronic conditions*				
No	9	50	0	0
Yes	9	50	1	1
*Patient registration with primary care provider*				
Not required or incentivised	6	33	0	0
Incentivised	5	28	1	1
Required	7	39	2	2
*Patient referral to secondary care*				
Not required or incentivised	3	17	0	0
Incentivised	5	28	1	1
Required	10	56	2	2
*Out of hours primary care: physicians rota*				
No	4	22	0	0
Yes	14	78	1	1
*Diabetes registry*				
No	10	56	0	0
Yes	8	44	1	1
*National guidelines for the management of diabetes*				
No	5	28	0	0
Yes	13	72	1	1
*Task-shifting from physicians to nurses in primary care*				
None	6	33	0	0
Limited	8	45	1	1
Advanced	4	22	2	2

^a^Number of countries = 18 and number of time periods = 3–7

### Country-level efficiency

Six variables showed a statistically significant association with efficiency: the prevalence of diabetes, obesity, smoking, alcohol consumption, education and income (Supplementary file, Table S3). We estimated a model for each of these and we show the results in Table 2. Efficiency scores lay within the range of zero to one (but scores were less than one due to bootstrapping), with a higher score indicating higher efficiency. For example, in the baseline model, Austria and Germany had the lowest efficiency scores, while Italy and the United Kingdom (UK) had the highest scores. These differences are likely driven by differences in diabetes admission and amputation rates, which were comparatively high for Austria and Germany but relatively low for Italy and the UK (Supplementary file, Figures S2 and S3).

**Table 2:** Country-level efficiency, average scores 2010–2016

Country	Baseline model	Baseline model with diabetes prevalence	Baseline model with alcohol	Baseline model with obesity	Baseline model with smoking	Baseline model with education	Baseline model with income
Austria	0.21	0.21	0.52	0.21	0.53	0.21	0.21
Belgium	0.58	0.57	0.70	0.61	0.78	0.58	0.57
Denmark	0.33	0.33	0.41	0.33	0.34	0.33	0.34
Finland	0.68	0.65	0.71	0.70	0.68	0.67	0.67
France	0.58	0.58	0.85	0.58	0.82	0.57	0.57
Germany	0.26	0.25	0.45	0.27	0.45	0.26	0.26
Ireland	0.76	0.75	0.85	0.73	0.82	0.77	0.76
Italy	0.81	0.77	0.85	0.80	0.84	0.80	0.78
Latvia	0.50	0.50	0.72	0.49	0.79	0.50	0.77
Lithuania	0.42	0.42	0.80	0.40	0.62	0.42	0.75
Luxembourg	0.37	0.36	0.68	0.38	0.39	0.36	0.37
Netherlands	0.80	0.80	0.87	0.80	0.85	0.79	0.81
Norway	0.57	0.57	0.60	0.58	0.59	0.57	0.58
Poland	0.74	0.74	0.82	0.74	0.79	0.74	0.75
Portugal	0.56	0.65	0.63	0.56	0.57	0.73	0.81
Spain	0.80	0.77	0.83	0.77	0.79	0.74	0.78
Sweden	0.75	0.75	0.79	0.75	0.78	0.76	0.76
United Kingdom	0.83	0.74	0.83	0.74	0.84	0.79	0.82

**Fig. 2:**
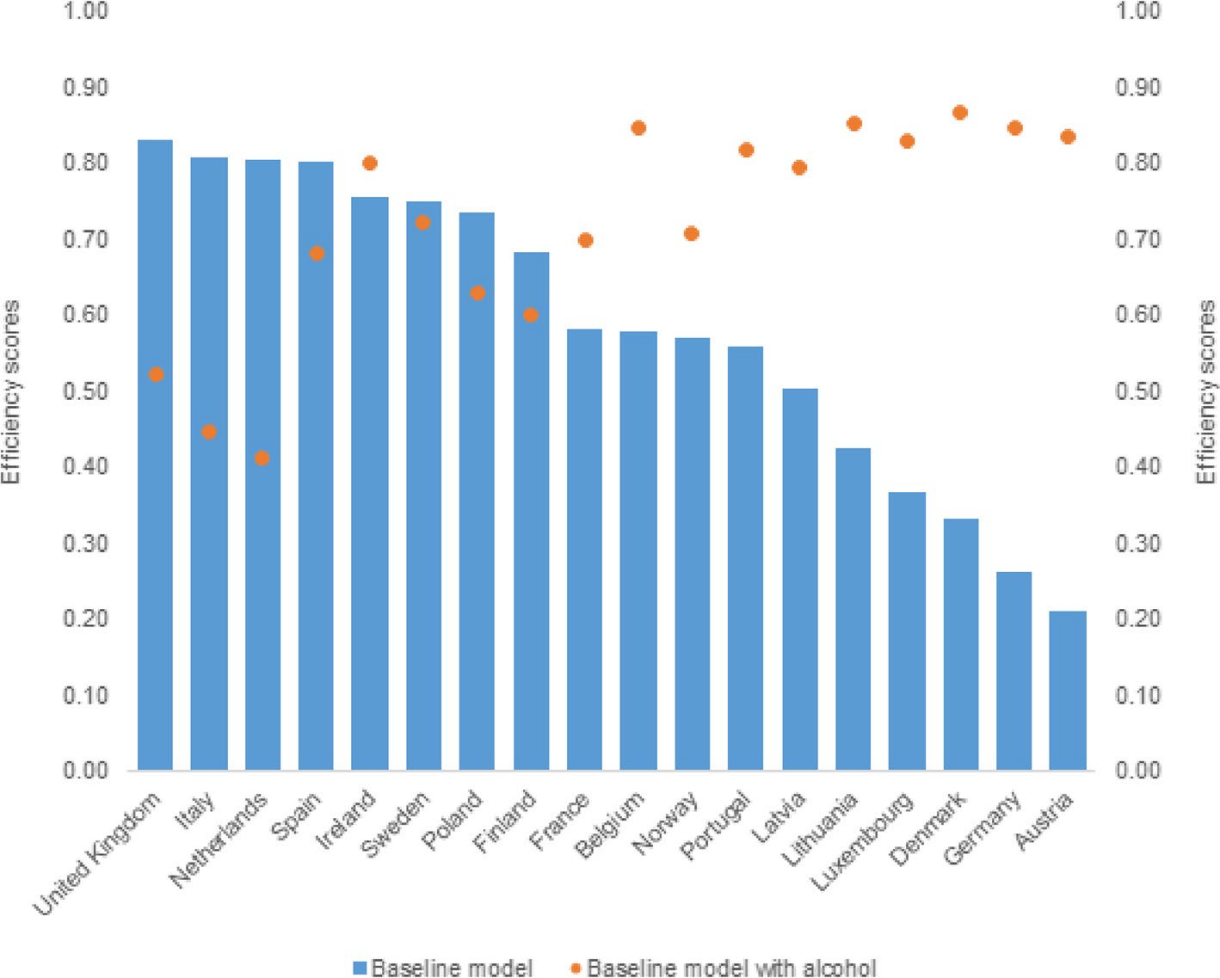
Efficiency scores from baseline model and model adjusted for alcohol consumption, average 2010–2016

Adjusting for different population characteristics improved the efficiency of countries although patterns varied. Most countries saw an improvement in efficiency scores after adjustment for alcohol consumption and smoking (Fig. 2). Portugal was the only country where adjusting for diabetes prevalence increased the primary care efficency score (from 0.56 to 0.65) as did adjusting for education. These observations are perhaps not suprising, since, for example, Portugal had the second-highest prevalence of diabetes and the lowest proportion of people with educational attainment to upper secondary level (Supplementary file 2 Figures S4 and S9). Similarly, levels of alcohol and tobacco use have traditionally been high in countries such as Austria, France, Germany, Lithuania and Luxembourg (Supplementary file Figures S5 and S7). Adjustment for income improved the efficiency score for several countries, most notably Latvia, Lithuania and Portugal. Adjustment for obesity changed efficiency scores only marginally, except in the United Kingdom, which had the highest level of obesity (26%) in the sample (Figure S6 in Supplementary file).

While the inclusion of different population characteristics changed country rankings to some extent, the Spearman rank correlations were relatively high (ranging from 0.63 to 0.97; also Table S4 in Supplementary file), suggesting a good degree of internal validity and relatively consistent country rankings across the models.

### Associations of efficiency and health system characteristics

Table 3 shows the results of the regression analysis investigating associations between health system characteristics and primary care efficiency across countries (see Table S5 in Supplementary file for quantitative results).

**Table 3:** Association of efficiency with health system characteristics

Health system characteristics	Baseline model	Baseline model with diabetes prevalence	Baseline model with alcohol	Baseline model with obesity	Baseline model with smoking	Baseline model with education	Baseline model with income
Curative care beds, per 1,000 population	-	-	-	-	-	-	-
Availability and use of Electronic Health Records (EHR) by GPs	+	+	n.s	+	n.s	+	n.s
Bonus payment: Yes (Reference = No)	+	+	+	+	+	+	+
Nurse-led follow-up: Yes (Reference = No)	+	+	+	+	+	+	+
Registration: Incentive (Reference = No incentive or obligation)	-	-	-	-	-	-	-
Registration: Required (Reference = No incentive or obligation)	n.s	n.s	n.s	n.s	n.s	n.s	+
Referral: Incentive (Reference = No incentive or obligation)	+	+	+	+	+	+	+
Referral: Required (Reference = No incentive or obligation)	+	+	+	+	+	+	+
Out of hours primary care: physicians rota: Yes (Reference = No)	-	-	n.s	-	n.s	-	-
Registry: Yes (Reference = No)	n.s	n.s	-	n.s	-	n.s	n.s
Guidelines: Yes (Reference = No)	n.s	+	n.s	n.s	n.s	n.s	n.s
Task-shifting: Limited (Reference = No task-shifting)	+	+	n.s	+	+	+	+
Task-shifting: Extensive (Reference = No task-shifting)	+	+	+	+	+	+	+

Note: + : positive statistically significant association, -: negative statistically significant association, n.s.: not statistically significant

We found that bonus payments for the prevention and management of chronic conditions and nurse-led follow-up were positively associated with efficiency in all models as was an incentive or requirement for primary care referral to specialist care. Task-shifting from physicians to nurses in primary care was positively associated with efficiency in all models except for limited task-shifting in the model adjusted for alcohol. There was also a positive association with efficiency for the availability of evidence-based national guidelines for diabetes, but only in the model adjusted for diabetes prevalence. Similarly, positive associations with efficiency for the availability and use of EHR by GPs were found for the baseline model and the models adjusting for diabetes prevalence, obesity and education. Conversely, higher rates of curative care beds were associated with lower efficiency in all models, as were incentives for patients to register with a primary care provider. The existence of a diabetes registry was negatively associated with efficiency in the models that included alcohol consumption and smoking. There was a negative association between efficiency and out-of-hours primary care provided by a rota of physicians in all models except for the models that adjusted for alcohol consumption and smoking.

## Discussion

In this paper, we investigated the efficiency of primary care systems in European countries and explored the associations between efficiency and health system characteristics. Primary care efficiency scores improved when a range of population characteristics were taken into account. We found that bonus payments, nurse-led follow-up of people with chronic conditions, and an incentive or requirement for patients to have a referral from primary to specialist care were associated with increased efficiency whereas the number of curative care beds and incentives for patients to register with a primary care provider reduced efficiency. For other health system variables, associations were less consistent.

Our finding that bonus payments were associated with higher efficiency aligns with other evidence suggesting that incentive payments in primary care, such as pay-for-performance schemes targeted at the management of chronic conditions, is associated with reduced resource use [58, 59] and gains in efficiency [60]. Likewise, the positive association between nurse-led follow-up of people with chronic conditions and efficiency, and task-shifting from physicians to nurses in primary care is also reflected in the wider literature suggesting that substitution of physicians by nurses in primary care can have a positive effect on health outcomes and patient satisfaction, although the effect on costs, health system outcomes, and quality of life is less conclusive [61–64]. Similarly, the association of a requirement to obtain a primary care referral to specialist care with higher efficiency aligns with previous studies [65, 66] that reported higher efficiency scores for OECD countries that had primary care gatekeeping arrangements in place.

Somewhat counterintuitively, evidence of an association between patient registration with a primary care provider and efficiency was mixed, with compulsory registration significantly positive only in the model that adjusted for income while voluntary registration using incentives was negatively associated with primary care efficiency in all models. Patient registration has been linked to enhancing care continuity and coordination [67], which, in turn, has been linked to improved patient outcomes [68] and lower service use and cost [69]. However, the nature and extent of how countries define and implement ‘patient registration’ varies substantially [70], and it is likely that the variable as conceptualized in the data source [44] used in this study captures some other mechanism that would explain our finding.

We also found some evidence that EHR availability and use may be associated with improved efficiency, although this applied to certain models in our study only. There is limited evidence, mostly from the United States, which points to the potential of EHR to increase efficiency in some contexts [71] while other studies have highlighted the negative impacts of inadequate design of EHR systems [72, 73].

A higher rate of curative care beds was associated with reduced efficiency. This finding is perhaps unsurprising as hospital beds built are likely to be used (‘Roemer’s Law’ [74–77]) although the relationship between hospital bed capacity and use is more complicated. For example, in an international comparative study Van Loenen et al. [78] found hospital bed supply to be strongly associated with admission rates for uncontrolled diabetes and long-term complications. They also highlighted the possibility of reverse causation, finding that countries that had a stronger primary care orientation also had lower hospital bed supply. Moreover, the price of hospital services varies widely across countries [79], which may have implications for efficiency.

We further found a negative association between efficiency and the organisation of out-of-hours primary care using a rota of physicians in all models except those that adjusted for alcohol consumption and smoking. A physician rota for out-of-hours primary care was the most common organisational model in our sample. Alternative approaches such as general practice co-operatives may be more efficient [80], but this model was not widespread and there may be insufficient statistical power to detect a positive association. A recent review [81] of national diabetes registries found that most registries served to monitor and improve the quality of diabetes care and that national registries may also help to achieve efficiency gains by identifying the causes of variation in outcomes. We did not find evidence to support this observation.

The study period covers six years (2010–2016), which coincided with primary care reform efforts in several countries that may be associated with efficiency. For example, in 2010, the Netherlands introduced a bundled payment for diabetes care provided in primary care settings. Evaluations showed that the reform led to improved care coordination and adherence to quality guidelines, improvements in clinical outcomes, and a reduction in the use of specialist care and associated costs [82]. Since the introduction of a new payment system for GPs in 2015, the bundled payment accounts for around 15% of GP income [83]. Similarly, Denmark introduced a bundled payment system in primary care for diabetes patients in 2007, but this was discontinued in 2014 due to low participation by GPs [84]. Evaluations have found that bundled payment models were associated with increased efficiency compared to separate payment for different services [85]. Therefore, we might expect that bundled payment would be positively associated with primary care efficiency. However, we are unable to test this hypothesis based on available data.

The implementation of austerity measures, following the 2007–08 financial crisis and subsequent global recession, may have affected primary (and secondary) care access and efficiency. However, it is difficult to investigate these changes given the diversity of responses across countries, encompassing changes to public funding, health coverage and health service planning, purchasing and delivery [86]. Additionally, an examination of the relationship between efficiency and quality regulations and regulatory actors was beyond the scope of this study.

While we included a variable measuring task-shifting from physicians to nurses in primary care, we did not consider the substitution of specialist care to primary care. A review of interventions involving the transfer of (elements of) services from specialist to primary care found some evidence of a reduction in the utilisation of specialist care but a lack of information on costs [87]. Evidence suggests that the relocation of specialists to primary care settings is associated with shorter waiting lists and times and improved patient satisfaction [88] as well as lower costs [89].

Not all countries in our study provide universal access to primary care. In Ireland, eligibility for free primary care services is based on age and income and less than half of the population meet the relevant criteria. Evidence suggests that people not eligible for free primary care are more likely to report unmet need for health care [90], and to forgo preventative [91], and chronic care [92]. While Irish government policy has prioritised universal primary care, modelling suggests that significant numbers of additional GPs would be needed to meet the increased demand arising from the introduction of universal primary care. One proposed solution to address the potential shortage of GPs is increased nurse substitution [93] and our findings of a positive relationship between task-shifting and efficiency would lend support to this policy.

While the time period of our study does not cover the COVID-19 pandemic, some of our results have relevance for the changes in health care delivery that were adopted in response to the pandemic. For example, the use of digital health tools increased substantially during the pandemic [94]. We found that the availability and use of one such tool (EHR) was associated with increased efficiency. A key finding of our study, namely that task-shifting from physicians to nurses in primary care was associated with increased efficiency, is very likely to remain significant given the continued efforts of countries to move to more systematic use of the non-physician workforce in primary care.

### Strengths and limitations

We used two indicators on the quality of primary care for diabetes, admissions and lower extremity amputation for diabetes. These are widely used in health system performance comparisons as indicators of the quality of diabetes care [95] and have also been used in a previous study [96] measuring the efficiency of diabetes care at a national level. National studies have also used more refined measures of diabetes care including diabetes-related medication [97, 98], the number of diabetic patients with a complete diabetes annual review [97] and a composite indicator of diabetes prevention and quality [96]. However, data on such indicators across countries and over time are currently unavailable. While focusing on a single condition facilitated the identification of appropriate outputs and cross-country comparisons, it is important to note that it does not reflect the wide range of activity undertaken in primary care and therefore our results would not be representative of primary care as a whole. Nevertheless, our results on the positive relationship between certain health system characteristics and efficiency may be relevant for other chronic conditions managed in primary care. Bonus payment and nurse-led follow-up are measured in relation to chronic illness in general and not specifically diabetes. Similarly, an incentive or requirement to receive a referral from primary to specialist care, and task-shifting from physicians to nurses are not restricted to a certain disease or patient population. While the OECD collects data on generalist medical practitioners according to a standardized definition, countries may differ in the extent to which their national data collection systems adhere to this definition, which may contribute to some of the differences across countries. As highlighted in our conceptual framework, nurses and other health care professionals play an important role in the care of people with diabetes, including in primary care settings. The OECD Health Statistics database includes data on nurses and pharmacists but does not distinguish between care settings. The lack of comparable data on nurses and other health professionals working in primary care settings, across countries and over time should be addressed in international databases. The exclusion of other primary care professions as inputs in the DEA models may have led to potential bias arising from the underestimation of the efficiency estimates [99, 100]. Many countries are implementing new models of delivering primary care using a team-based approach [11] and research suggests that collaborative and team-based care may improve clinical outcomes for diabetes care [101], and reduce the use of acute care for patients with chronic illness [102]. However, heterogeneity in the composition of primary care teams and the lack of comparable data across countries restricted consideration of the relationship between team-based care and efficiency. We pooled data over time in order to increase the sample size and the reliability of our results but a potential drawback is that we overlook change in efficiency over time. Nevertheless, our approach is in line with previous studies in the healthcare context [3, 103, 104].

## Conclusions

This study contributes to the evidence base on measuring the efficiency of primary care systems across countries and their relevant correlates and explanatory factors. Differences in efficiency across countries were driven, to a considerable degree, by population differences but our findings also suggest that countries might achieve greater efficiency by implementing systematic efforts for enhancing the management of chronic diseases in primary care supported by bonus payments, nurse-led patient follow-up, and appropriate referral systems. There is a need to improve and extend current data collection in order to produce a set of core indicators that would enable more comprehensive assessments of primary care efficiency across countries. Future qualitative, in-depth country case study research could also provide useful additional insights into the features of those countries that this study revealed as better performers on efficiency.

### Supplementary Information

Below is the link to the electronic supplementary material.

**Additional file 1: Table S1.** Input, outputs and population characteristics variables. **Table S2.** Health system characteristics variables. **Table S3.** Association between efficiency and population characteristics. **Table S4.** Correlations between models. **Table S5.** Truncated regression results. **Table S6.** Results of log likelihood ratio tests for inclusion of year variables (time fixed effects). **Figure S1.** Generalist medical practitioners, per 1,000 population, average 2010-2016. **Figure S2.** Diabetes hospital admissions per 100,000 population, average 2010-2016. **Figure S3.** Admission based diabetes lower extremity amputation, per 100,000 population, average 2010-2016. **Figure S4.** Diabetes prevalence, percentage of population (age-standardised) , average 2010-2016. **Figure S5.** Alcohol consumption, litres per capita, average 2010-2016. **Figure S6.** Prevalence of obesity among adults aged 18 years and over, (%), average 2010-2016. **Figure S7.** Smoking, percentage of population aged 15+, average 2010-2016. **Figure S8.** Severe material deprivation, percentage of population, average 2010-2016. **Figure S9.** Upper secondary level education, % of population aged 25-64 years, average 2010-2016. **Figure S10.** Income per capita, US$ Purchasing Power Parity, average 2010-2016. **Figure S11.** Percentage of total unemployed population unemployed for one year or more, average 2010-2016. **Figure S12.** Curative care beds, per 1,000 population, average 2010-2016. **Figure S13.** Availability and use of Electronic Health Records by GPs, average 2010-2016.

## Data Availability

The datasets used and analysed during the current study are available from the following websites: OECD Health Statistics: https://stats.oecd.org/Index.aspx?ThemeTreeId=9 Institute of Health Metrics and Evaluation: https://www.healthdata.org/ WHO Global Health Observatory: https://www.who.int/data/gho The World Bank: https://data.worldbank.org/ Eurostat: https://ec.europa.eu/eurostat/data/database OECD Education at a Glance: https://www.oecd-ilibrary.org/education/data/oecd-education-statistics_edu-data-en

## References

[1] Papanicolas I, Smith PC (2013). Health system performance comparison. An agenda for policy, information and research.

[2] Cylus J, Papanicolas I, Smith PC. Health system efficiency: How to make measurement matter for policy and management: European Observatory on Health Systems and Policies; 2016. 28783269

[3] Varabyova Y, Schreyögg J (2013). International comparisons of the technical efficiency of the hospital sector: panel data analysis of OECD countries using parametric and non-parametric approaches. Health Policy.

[4] Hollingsworth B (2008). The measurement of efficiency and productivity of health care delivery. Health Econ.

[5] Kohl S, Schoenfelder J, Fügener A, Brunner JO (2019). The use of Data Envelopment Analysis (DEA) in healthcare with a focus on hospitals. Health Care Manag Sci.

[6] Amado CA, Santos SP (2009). Challenges for performance assessment and improvement in primary health care: the case of the Portuguese health centres. Health Policy.

[7] Filipe Amado CA, Dyson RG (2008). On comparing the performance of primary care providers. Eur J Oper Res.

[8] Neri M, Cubi-Molla P, Cookson G (2022). Approaches to measure efficiency in primary care: a systematic literature review. Appl Health Econ Health Policy.

[9] World Health Organisation, editor Declaration of Astana: from Alma-Ata towards universal health coverage and the Sustainable Development Goals. Global Conference on Primary Health Care; 2018; Astana, Kazakhstan.

[10] World Health Organisation. Building the economic case for primary health care: a scoping review. World Health Organisation; 2018.

[11] OECD (2020). Realising the Potential of Primary Health Care.

[12] Nolte E, Knai C, Saltman RB (2014). Assessing chronic disease management in European health systems: Concepts and approaches.

[13] European Commission (2018). A new drive for primary care in Europe: rethinking the assessment tools and methodologies. Report of the expert group on health systems performance assessment.

[14] Zakowska I, Godycki-Cwirko M (2020). Data envelopment analysis applications in primary health care: a systematic review. Fam Pract.

[15] Pelone F, Kringos DS, Romaniello A, Archibugi M, Salsiri C, Ricciardi W (2015). Primary care efficiency measurement using data envelopment analysis: a systematic review. J Med Syst.

[16] Pelone F, Kringos DS, Spreeuwenberg P, De Belvis AG, Groenewegen PP. How to achieve optimal organization of primary care service delivery at system level: lessons from Europe. International journal for quality in health care : journal of the International Society for Quality in Health Care. 2013;25(4):381–93. 10.1093/intqhc/mzt020 23407822

[17] OECD/European Union (2020). Health at a Glance: Europe 2020: state of health in the EU cycle.

[18] Nolte E, Bain C, McKee M (2006). Diabetes as a tracer condition in international benchmarking of health systems. Diabetes Care.

[19] European Commission (2014). Expert panel on effective ways of investing in health (EXPH) Definition of a frame of reference in relation to primary care with a special emphasis on financing systems and referral systems.

[20] Brown AF, Ettner SL, Piette J, Weinberger M, Gregg E, Shapiro MF (2004). Socioeconomic position and health among persons with diabetes mellitus: a conceptual framework and review of the literature. Epidemiol Rev.

[21] World Health Organization (2010). Monitoring the building blocks of health systems: a handbook of indicators and their measurement strategies.

[22] World Health Organisation Regional Office for Europe (2019). Indicator Passport WHO European Primary Health Care, Impact, Performance and Capacity Tool (PHC-IMPACT).

[23] World Health Organisation (2007). Everybody's business : strengthening health systems to improve health outcomes : WHO’s framework for action.

[24] OECD (2021). OECD Health Statistics 2021.

[25] Jacobs R, Smith PC, Street A (2006). Measuring efficiency in health care: analytic techniques and health policy.

[26] GBD Results Tool [Internet]. IHME, University of Washington. 2022. Available from: https://ghdx.healthdata.org/gbd-results-tool.

[27] Global Health Observatory [Internet]. World Health Organisation. 2022. Available from: https://www.who.int/data/gho/data/indicators.

[28] World Bank Open Data [Internet]. The World Bank. 2022. Available from: https://data.worldbank.org/.

[29] Database [Internet]. Eurostat. 2022. Available from: https://ec.europa.eu/eurostat/data/database.

[30] Adult education level (indicator). [Internet]. 2021. Available from: 10.1787/36bce3fe-en

[31] OECD Statistics [Internet]. OECD Publishing. 2022. Available from: https://stats.oecd.org/.

[32] OECD. Health at a Glance 2019. 2019.

[33] Jeffcoate WJ, van Houtum WH (2004). Amputation as a marker of the quality of foot care in diabetes. Diabetologia.

[34] Comino EJ, Harris MF, Islam MD, Tran DT, Jalaludin B, Jorm L (2015). Impact of diabetes on hospital admission and length of stay among a general population aged 45 year or more: a record linkage study. BMC Health Serv Res.

[35] Liu M, Zhang W, Yan Z, Yuan X (2018). Smoking increases the risk of diabetic foot amputation: a meta-analysis. Exp Ther Med.

[36] Ko KD, Kim BH, Park SM, In OhS, Um CS, Shin DW (2012). What are patient factors associated with the quality of diabetes care?: results from the Korean National Health and Nutrition Examination Survey. BMC Public Health.

[37] Hill J, Nielsen M, Fox MH (2013). Understanding the social factors that contribute to diabetes: a means to informing health care and social policies for the chronically ill. Perm J.

[38] Walker RJ, Gebregziabher M, Martin-Harris B, Egede LE (2014). Relationship between social determinants of health and processes and outcomes in adults with type 2 diabetes: validation of a conceptual framework. BMC Endocr Disord.

[39] Wolters RJ, Braspenning JCC, Wensing M (2017). Impact of primary care on hospital admission rates for diabetes patients: a systematic review. Diabetes Res Clin Pract.

[40] Hadad S, Hadad Y, Simon-Tuval T (2013). Determinants of healthcare system's efficiency in OECD countries. Eur J Health Econ: HEPAC Health Econ Prev Care.

[41] Lewis HF, Sexton TR (2004). Data envelopment analysis with reverse inputs and outputs. J Prod Anal.

[42] Scheel H (2001). Undesirable outputs in efficiency valuations. Eur J Oper Res.

[43] Codagnone C, Lupiañez-Villanueva F (2013). Benchmarking deployment of ehealth among general practitioners.

[44] Health Systems Characteristics [Internet]. OECD Publishing. Available from: https://www.oecd.org/els/health-systems/characteristics.htm.

[45] Chevreul K, Berg Brigham K, Durand-Zaleski I, Hernández-Quevedo C. France: Health system review. 2015. 26766545

[46] Sowada C, Sagan A, Kowalska-Bobko I, Badora-Musiał K, Bochenek T, Domagała A, et al. Poland: Health system review. 2019. 1–235. 31333192

[47] Sperre Saunes I, Karanikolos M, 2020; SA. Norway: Health system review. 2020. 32863241

[48] Noncommunicable diseases: National capacity [Internet]. World Health Organisation. 2022. Available from: https://www.who.int/data/gho/data/themes/topics/topic-details/GHO/ncd-national-capacity.

[49] Bała MM, Płaczkiewicz-Jankowska E, Leśniak W, Topór-Mądry R, Jankowski M, Grzeszczak W (2014). Management and treatment goals in Polish patients with type 2 diabetes of more than ten years' duration - results of ARETAEUS2-Grupa study. Endokrynol Pol.

[50] Maier CB, Aiken LH (2016). Task shifting from physicians to nurses in primary care in 39 countries: a cross-country comparative study. Eur J Public Health.

[51] Simar L, Wilson PW (2007). Estimation and inference in two-stage, semi-parametric models of production processes. J Econometr.

[52] Hollingsworth B. Health system efficiency: measurement and policy. In: Cylus J, Papanicolas I, Smith P, editors. Health system efficiency: How to make measurement matter for policy and management: European Observatory on Health Systems and Policies; 2016. p. 99–137. 28783269

[53] Banker RD, Charnes A, Cooper WW (1984). Some models for estimating technical and scale inefficiencies in data envelopment analysis. Manage Sci.

[54] Hollingsworth B, Smith P (2003). Use of ratios in data envelopment analysis. Appl Econ Lett.

[55] Simar L, Wilson PW (1998). Sensitivity analysis of efficiency scores: how to bootstrap in nonparametric frontier models. Manage Sci.

[56] R Core Team (2021). R: A language and environment for statistical computing.

[57] StataCorp (2021). Stata Statistical Software: Release 17.

[58] Harrison MJ, Dusheiko M, Sutton M, Gravelle H, Doran T, Roland M (2014). Effect of a national primary care pay for performance scheme on emergency hospital admissions for ambulatory care sensitive conditions: controlled longitudinal study. BMJ Brit Med J.

[59] Fiorentini G, Iezzi E, Lippi Bruni M, Ugolini C (2011). Incentives in primary care and their impact on potentially avoidable hospital admissions. Eur J Health Econ: HEPAC Health Econ Prev Care.

[60] Cashin C, Chi Y-L, Borowitz M. Lessons from the case study P4P programmes. In: Cashin C, Chi Y-L, Smith P, Borowitz M, Thomson S, editors. Paying for Performance in Health Care Implications for health system performance and accountability. UK: Open University Press; 2014.

[61] Martínez-González NA, Djalali S, Tandjung R, Huber-Geismann F, Markun S, Wensing M (2014). Substitution of physicians by nurses in primary care: a systematic review and meta-analysis. BMC Health Serv Res.

[62] Martínez-González NA, Tandjung R, Djalali S, Rosemann T (2015). The impact of physician-nurse task shifting in primary care on the course of disease: a systematic review. Hum Resour Health.

[63] Laurant M, van der Biezen M, Wijers N, Watananirun K, Kontopantelis E, van Vught AJ (2018). Nurses as substitutes for doctors in primary care. Cochrane Database Syst Rev..

[64] Winkelmann J, Williams GA, Rijken M, Polin K, Maier CB, Maier CB, Kroezen M, Wismar M, Busse R (2022). Chronic conditions and multimorbidity: skill-mix innovations for enhanced quality and coordination of care. Skill-mix Innovation, Effectiveness and Implementation: Improving Primary and Chronic Care. European Observatory on Health Systems and Policies.

[65] Blöndal B, Ásgeirsdóttir TL (2019). Costs and efficiency of gatekeeping under varying numbers of general practitioners. Int J Health Plann Manage.

[66] Bhat VN (2005). Institutional arrangements and efficiency of health care delivery systems. Eur J Health Econ: HEPAC Health Econ Prev Care.

[67] Boerma W, Dubois C-A, Saltman RB, Rico A, Boerma W (2006). Mapping primary care across Europe. Primary care in the driver's seat? Organisational reform in European primary care.

[68] van Walraven C, Oake N, Jennings A, Forster AJ (2010). The association between continuity of care and outcomes: a systematic and critical review. J Eval Clin Pract.

[69] Nicolet A, Al-Gobari M, Perraudin C, Wagner J, Peytremann-Bridevaux I, Marti J (2022). Association between continuity of care (COC), healthcare use and costs: what can we learn from claims data? a rapid review. BMC Health Serv Res.

[70] Marchildon GP, Brammli-Greenberg S, Dayan M, De Belvis AG, Gandré C, Isaksson D (2021). Achieving higher performing primary care through patient registration: a review of twelve high-income countries. Health Policy.

[71] Bae J, Encinosa WE (2016). National estimates of the impact of electronic health records on the workload of primary care physicians. BMC Health Serv Res.

[72] Kroth PJ, Morioka-Douglas N, Veres S, Babbott S, Poplau S, Qeadan F (2019). Association of electronic health record design and use factors with clinician stress and burnout. JAMA Netw Open.

[73] Menon S, Murphy DR, Singh H, Meyer AN, Sittig DF (2016). Workarounds and test results follow-up in electronic health record-based primary care. Appl Clin Inform.

[74] Roemer MI (1961). Bed supply and hospital utilization: a natural experiment. Hospitals.

[75] Taroni F (2001). Roemer's effect reconsidered. J Health Serv Res Policy.

[76] Delamater PL, Messina JP, Grady SC, WinklerPrins V, Shortridge AM (2013). Do more hospital beds lead to higher hospitalization rates? a spatial examination of Roemer’s law. PLoS ONE.

[77] Shwartz M, Peköz EA, Labonte A, Heineke J, Restuccia JD (2011). Bringing responsibility for small area variations in hospitalization rates back to the hospital: the propensity to hospitalize index and a test of the Roemer's law. Med Care.

[78] Van Loenen T, Faber MJ, Westert GP, Van den Berg MJ (2016). The impact of primary care organization on avoidable hospital admissions for diabetes in 23 countries. Scand J Prim Health Care.

[79] Lorenzoni L, Dougherty S (2022). Understanding differences in health care spending: a comparative study of prices and volumes across OECD countries. Health Serv Insights.

[80] Huibers L, Giesen P, Wensing M, Grol R (2009). Out-of-hours care in western countries: assessment of different organizational models. BMC Health Serv Res.

[81] Bak JCG, Serné EH, Kramer MHH, Nieuwdorp M, Verheugt CL (2021). National diabetes registries: do they make a difference?. Acta Diabetol.

[82] Struijs JN, Drewes HW, Heijink R, Baan CA, Amelung V, Stein V, Goodwin N, Balicer R, Nolte E, Suter E (2017). Netherlands: the potentials of integrating care via payment reforms. Handbook Integrated Care.

[83] Schut FT, Varkevisser M (2017). Competition policy for health care provision in the Netherlands. Health Policy.

[84] OECD. Better ways to pay for health care. 2016.

[85] Feldhaus I, Mathauer I (2018). Effects of mixed provider payment systems and aligned cost sharing practices on expenditure growth management, efficiency, and equity: a structured review of the literature. BMC Health Serv Res.

[86] Thomson S, Figueras J, Evetovits T, Jowett M, Mladovsky P, Maresso A, et al. Economic crisis, health systems and health in Europe: impact and implications for policy. World Health Organization (acting as the host organization for aso, the European Observatory on Health Systems and Policies, editor. England: Open University Press; 2015. 28837306

[87] Winpenny EM, Miani C, Pitchforth E, King S, Roland M (2017). Improving the effectiveness and efficiency of outpatient services: a scoping review of interventions at the primary-secondary care interface. J Health Serv Res Policy.

[88] van Hoof SJM, Quanjel TCC, Kroese M, Spreeuwenberg MD, Ruwaard D (2019). Substitution of outpatient hospital care with specialist care in the primary care setting: a systematic review on quality of care, health and costs. PLoS ONE.

[89] van den Bogaart EHA, Kroese MEAL, Spreeuwenberg MD, Ruwaard D, Tsiachristas A (2021). Economic evaluation of new models of care: does the decision change between cost-utility analysis and multi-criteria decision analysis?. Value Health.

[90] Connolly S, Wren M-A (2017). Unmet healthcare needs in Ireland: analysis using the EU-SILC survey. Health Policy.

[91] Mc Hugh SM, Browne J, O’Neill C, Kearney PM (2015). The influence of partial public reimbursement on vaccination uptake in the older population: a cross-sectional study. BMC Public Health.

[92] Murphy CM, Kearney PM, Shelley EB, Fahey T, Dooley C, Kenny RA (2016). Hypertension prevalence, awareness, treatment and control in the over 50s in Ireland: evidence from the Irish longitudinal study on ageing. J Public Health.

[93] Teljeur C, Thomas S, O'Kelly FD, O'Dowd T (2010). General practitioner workforce planning: assessment of four policy directions. BMC Health Serv Res.

[94] Fahy N, Williams GA (2021). COVID-19 Health System Response Monitor Network. Use of digital health tools in Europe: before, during and after COVID-19.

[95] OECD (2021). Health at a Glance 2021: OECD Indicators.

[96] Ramalho A, Souza J, Castro P, Lobo M, Santos P, Freitas A. Portuguese primary healthcare and prevention quality indicators for diabetes mellitus – a data envelopment analysis. Int J Health Policy Manage. 2021. 10.34172/ijhpm.2021.76 PMC9808229 34380198

[97] Amado CAF, Dyson RG (2009). Exploring the use of DEA for formative evaluation in primary diabetes care: an application to compare English practices. J Operational Res Soc.

[98] Testi A, Fareed N, Ozcan YA, Tanfani E (2013). Assessment of physician performance for diabetes: a bias-corrected data envelopment analysis model. Qual Prim Care.

[99] Smith P (1997). Model misspecification in data envelopment analysis. Ann Oper Res.

[100] Galagedera DUA, Silvapulle P (2003). Experimental evidence on robustness of data envelopment analysis. J Oper Res Soc.

[101] Lee JK, McCutcheon LRM, Fazel MT, Cooley JH, Slack MK (2021). Assessment of interprofessional collaborative practices and outcomes in adults with diabetes and hypertension in primary care: a systematic review and meta-analysis. JAMA Netw Open.

[102] Meyers DJ, Chien AT, Nguyen KH, Li Z, Singer SJ, Rosenthal MB (2019). Association of team-based primary care with health care utilization and costs among chronically ill patients. JAMA Intern Med.

[103] Lepine A, Vassall A, Chandrashekar S (2015). The determinants of technical efficiency of a large scale HIV prevention project: application of the DEA double bootstrap using panel data from the Indian Avahan. Cost Effective Resource Allocation: C/E.

[104] Ravangard R, Hatam N, Teimourizad A, Jafari A (2014). Factors affecting the technical efficiency of health systems: a case study of Economic Cooperation Organization (ECO) countries (2004–10). Int J Health Policy Manag.

