# Supplementary material for: Exploring the association between primary care efficiency and health system characteristics across European countries: a two-stage data envelopment analysis
Source: BMC Health Serv Res. 2023 Dec 4;23:1348. doi: 10.1186/s12913-023-10369-y (PMC10694950; doi:10.1186/s12913-023-10369-y)
Supplement: Supplementary file 1 — Additional file 1: Table S1. Input, outputs and population characteristics variables. Table S2. Health system characteristics variables. Table S3. Association between efficiency and population characteristics. Table S4. Correlations between models. Table S5. Truncated regression results. Table S6. Results of log likelihood ratio tests for inclusion of year variables (time fixed effects). Figure S1. Generalist medical practitioners, per 1,000 population, average 2010-2016. Figure S2. Diabetes hospital admissions per 100,000 population, average 2010-2016. Figure S3. Admission based diabetes lower extremity amputation, per 100,000 population, average 2010-2016. Figure S4. Diabetes prevalence, percentage of population (age-standardised) , average 2010-2016. Figure S5. Alcohol consumption, litres per capita, average 2010-2016. Figure S6. Prevalence of obesity among adults aged 18 years and over, (%), average 2010-2016. Figure S7. Smoking, percentage of population aged 15+, average 2010-2016. Figure S8. Severe material deprivation, percentage of population, average 2010-2016. Figure S9. Upper secondary level education, % of population aged 25-64 years, average 2010-2016. Figure S10. Income per capita, US$ Purchasing Power Parity, average 2010-2016. Figure S11. Percentage of total unemployed population unemployed for one year or more, average 2010-2016. Figure S12. Curative care beds, per 1,000 population, average 2010-2016. Figure S13. Availability and use of Electronic Health Records by GPs, average 2010-2016. [file 12913_2023_10369_MOESM1_ESM.docx]

**Article title:** Exploring the Association between Primary Care Efficiency and Health System Characteristics across European Countries: A Two-Stage Data Envelopment Analysis

**Authors’ information**:

Valerie Moran^1,2^, Marc Suhrcke^1,2,3^, Ellen Nolte^4^

^1^ Socio-Economic and Environmental Health and Health Services Research Group, Department of Precision Health, Luxembourg Institute of Health, Strassen, Luxembourg (corresponding author: valerie.moran@lih.lu)

^2^ Socio-Economic and Environmental Health and Health Services Research Group, Living Conditions Department, Luxembourg Institute of Socio-Economic Research, Esch/Belval, Luxembourg (corresponding author: [valerie.moran@liser.lu](mailto:valerie.moran@liser.lu))

^3^ Centre for Health Economics, University of York, York, UK

^4^London School of Hygiene and Tropical Medicine, London, UK

**Supplementary file**

**Table S1. Input, outputs and population characteristics variables**

| Indicator | Source | Definition | Missing data | Variable construction for analysis |
| --- | --- | --- | --- | --- |
| Input | |  |  |  |
| Generalist medical practitioners, per 1000 population | OECD Health Statistics 2021 | Inclusion  - General practitioners  - District medical doctors - therapists  - Family medical practitioners  - Primary health care physicians  - Medical doctors (general)  - Medical officers (general)  - Medical interns or residents specialising in general practice or without any area of specialisation  yet  Exclusion  - Paediatricians  - Obstetricians and gynaecologists  - Specialist physicians (internal medicine) | Finland: 2016;  Hungary (all years); Slovak Republic (all years) | None |
| Output | |  |  |  |
| Diabetes hospital admission, age-sex standardised rate per 100,000 population aged 15 and over | OECD Health Statistics 2021 | Numerator: acute hospital admissions with a principal diagnosis of diabetes. Excludes in-patient deaths, transfers in, maternal/neonatal cases, and same-day admissions.  Denominator: Population count. | Belgium: 2015; Estonia: 2010-2013;  France: 2016; Germany: 2010, 2012, 2014, 2016; Hungary: 2013-2016; Italy: 2016; Latvia: 2010; Lithuania: 2010-2011; Luxembourg: 2016; Netherlands: 2013; Portugal: 2010, 2012, 2014; Slovak Republic: 2010-2011; United Kingdom: 2010 | Use multiplicative inverse rate  Missing data for Germany are imputed based on simple average of 2009 and 2011 for 2010; 2011 and 2013 for 2012; 2013 and 2015 for 2014 and 2015 and 2017 for 2016.  Missing data for Portugal for 2014 is imputed based on simple average of 2013 and 2015. |

**Table S1. Input, outputs and population characteristics variables *continued***

| Indicator | Source | Definition | Missing data | Variable construction for DEA analysis |
| --- | --- | --- | --- | --- |
| Admission based diabetes lower extremity amputation, age-sex standardised rate per 100,000 population aged 15 and over | OECD Health Statistics 2021 | Numerator: acute hospital admissions with a procedure code of major lower extremity amputation in any field and a diagnosis code of diabetes in any field. Excludes transfers in, maternal/neonatal cases, cases with trauma diagnosis code, cases with tumour-related peripheral amputation code and same-day admissions.  Denominator: Population count. | Belgium, 2015-2016; Czech Republic (all years);  Estonia: 2010-2015; France: 2016; Germany: 2010, 2012, 2014, 2016; Italy: 2014-2016;  Latvia: 2010-2013;  Lithuania: 2010-2011;  Luxembourg: 2016  Netherlands: 2012-2013; Portugal: 2010-2012, 2014; Slovak Republic (all years);  Switzerland: 2010-2011, 2013-2014, 2016  United Kingdom: 2010 | Use multiplicative inverse rate  Missing data for Germany are imputed based on simple average of 2009 and 2011 for 2010; 2011 and 2013 for 2012; and 2013 and 2015 for 2014.  Missing data for Portugal for 2014 is imputed based on simple average of 2013 and 2015. |
| Population characteristics | |  |  |  |
| Diabetes prevalence, age-standardised, percentage of population | Institute of Health Metrics and Evaluation (IHME) | Age-standardised prevalence of diabetes mellitus (type 1 and 2), percentage of population | None | Use multiplicative inverse rate |
| Alcohol consumption, litres per capita | OECD Health Statistics 2021 | Litres per capita | None | Use multiplicative inverse rate |
| Prevalence of obesity among adults aged 18 years and over, (%) | WHO GHO | Body Mass Index (BMI) greater than or equal to 30 (age-standardized estimate) | None | Use multiplicative inverse rate |
| Smoking, percentage of population aged 15+ | The World Bank | Percentage of men and women aged 15 and over who currently smoke any tobacco product (excluding smokeless tobacco use) on a daily or non-daily basis. The rates are age-standardized. | None | Use multiplicative inverse rate |
| Severe material deprivation, percentage of population | Eurostat – EU-SILC | Percentage of population with an enforced lack of at least four out of nine material deprivation items in the 'economic strain and durables' dimension. | None | Not included as uncontrollable input |
| Upper secondary level education, % of population aged 25-64 years | OECD Education at a Glance 2021 | Percentage of 25-64 year-olds with upper secondary level education | None | Not applicable |
| Income per capita | OECD Statistics 2021 | Gross Domestic Product (GDP) per capita, US$ Purchasing Power Parity | None | Not applicable |
| Percentage of total unemployed population unemployed for one year or more | OECD Statistics 2021 | Percentage of total unemployed population aged 15 years and over, unemployed for one year or more | None | Not included as uncontrollable input |

**Table S2. Health system characteristics variables**

| Variable | Data Source | Missing data | Variable construction for analysis |
| --- | --- | --- | --- |
| Curative care beds, per 1,000 population | OECD Health Statistics 2021 (Data covers 2010-2016) | Denmark: missing data for 2012  No data for United Kingdom as it is not possible to separate curative care beds from beds for rehabilitation, long-term care and palliative care.  Used total hospital beds for UK | Missing data for Denmark is imputed based on simple average 2011 and 2013 |
| Electronic Health Records (EHR): a sample of 9,116 GPs that have and use a computer were asked about the availability and use of 25 Electronic Health Record (EHR) functionalities. Based on the responses, a composite indicator was constructed ranging from 0 to 4: 1=Not available, 2=Available but not used, 3=Occasional use, 4 Routine use | European Commission (2013) “Benchmarking Deployment of eHealth among General Practitioners (2013)” Final Report | None | Continuous variable ranging from 1 to 4 |
| Primary care providers receive bonus payment for achieving targets relating to the prevention and management of chronic diseases (*bonus payment*) | OECD Health System Characteristics Survey 2016 | None | Binary variable:  No=0, 1=Yes |
| A large majority (>75%) of nurses or assistants independently provide routine checks of chronically ill patients (*nurse-led follow-up*) | OECD Health System Characteristics Survey 2016 | None | Binary variable:  No=0, 1=Yes |
| Patients are required or encouraged to register with a primary care physician or practice (*registration*) | OECD Health System Characteristics Survey 2016 | None | 0=Obliged to register  1=Financial incentive to register  2=No incentive or obligation to register |
| Primary care physicians control access to specialist care (requirement for patients to obtain primary care referral to specialist care: *referral)* | OECD Health System Characteristics Survey 2016 | None | 0=Referral is required  1=Financial incentive to obtain referral  2=No requirement or incentive to obtain referral |
| Arrangements for out-of-hours primary care: group of physicians on a rota basis (*out of hours care, physicians rota)* | OECD Health System Characteristics Survey 2016 | France, Norway, Poland: Missing data replaced with information from European Observatory on Health Systems and Policies HiT report | Binary variable:  No=0, 1=Yes |
| Country has a diabetes registry (*registry: yes/no)* | WHO NCD Country Capacity Survey 2015 | None | No=0, 1=Yes |
| Government approved evidence-based national guidelines for the management of diabetes (*guidelines: yes/no*) | WHO NCD Country Capacity Survey 2015 | France, Poland: Missing data replaced with information from European Observatory on Health Systems and Policies HiT report 2015 for France and Bala et al. (2014) for Poland | No=0, 1=Yes |
| Task-shifting from physicians to nurses in primary care: Data from an international expert survey of 93 country experts in 39 countries, covering Europe, USA, Canada, Australia and New Zealand (response rate: 85.3%), plus a literature scoping review. Seven clinical activities including prescriptions, diagnosis, ordering of tests, panel of patients, treatment referral, and being first point of contact were considered. Task-shifting was measured by nurses undertaking: none, at least two (limited), or all seven (extensive) of the activities. The ‘extensive’ category also included education (usually up to Master’s level) as a Nurse Practitioner/Advanced Practice Nurse. | Maier, C.B., Aiken, L.H. (2016) “Task-shifting from physicians to nurses in primary care in 39 countries: a cross-country comparative study | None | 0=No task-shifting  1=Limited task-shifting  2=Extensive task-shifting |

**Table S3. Association between efficiency and population characteristics**

| **Variable, n=106** | **Coefficient** | **Standard Error** |
| --- | --- | --- |
| Diabetes prevalence, % | 0.05 | 0.02** |
| Smoking, % | -0.02 | 0.01** |
| Alcohol consumption, litres per capita | -0.06 | 0.01*** |
| Obesity, % of population aged 18 years and over | 0.04 | 0.01** |
| Deprivation, % of population | 0.01 | 0.01 |
| Income per capita, US$ per capita (log) | -0.20 | 0.08* |
| Percentage of total unemployed population unemployed for one year or more | 0.00 | 0.00 |
| Upper second level education, % of population aged 25-64 years | -0.01 | 0.00*** |

**Table S4. Correlations between models**

|  | **Baseline model** | **Baseline model with diabetes prevalence** | **Baseline model with alcohol** | **Baseline model with obesity** | **Baseline model with smoking** | **Baseline model with education** | **Baseline model with income** |
| --- | --- | --- | --- | --- | --- | --- | --- |
| **Baseline Model** | 1 |  |  |  |  |  |  |
| **Baseline model with diabetes prevalence** | 0.97 | 1 |  |  |  |  |  |
| **Baseline model with alcohol** | 0.71 | 0.69 | 1 |  |  |  |  |
| **Baseline model with obesity** | 0.97 | 0.97 | 0.72 | 1 |  |  |  |
| **Baseline model with smoking** | 0.79 | 0.76 | 0.79 | 0.76 | 1 |  |  |
| **Baseline model with education** | 0.95 | 0.95 | 0.68 | 0.94 | 0.75 | 1 |  |
| **Baseline model with income** | 0.86 | 0.86 | 0.63 | 0.82 | 0.70 | 0.87 | 1 |

Note: all correlations are significant at p<0.000

**Table S5. Truncated regression results**

|  | **Baseline model** | **Baseline model with diabetes prevalence** | **Baseline model with alcohol** | **Baseline model with obesity** | **Baseline model with smoking** | **Baseline model with education** | **Baseline model with income** |
| --- | --- | --- | --- | --- | --- | --- | --- |
| **Variable** | **Coefficient (SE)** | **Coefficient (SE)** | **Coefficient (SE)** | **Coefficient (SE)** | **Coefficient (SE)** | **Coefficient (SE)** | **Coefficient (SE)** |
| Curative care beds, per 1,000 population | -0.11 (0.01)*** | -0.11 (0.01)*** | -0.06 (0.02)*** | -0.1 (0.01)*** | -0.06 (0.01)*** | -0.11 (0.01)*** | -0.10 (0.02)*** |
| Constant | 1.02 (0.05)*** | 0.98 (0.04)*** | 0.98 (0.06)*** | 0.97 (0.04)*** | 0.94 (0.06)*** | 1 (0.04)*** | 1.03 (0.06)*** |
| Availability and use of Electronic Health Records (EHR) by GPs | 0.13 (0.04)** | 0.1 (0.04)* | -0.05 (0.04) | 0.12 (0.04)** | 0.03 (0.04) | 0.11 (0.04)** | -0.08 (0.05) |
| Constant | 0.22 (0.13) | 0.29 (0.12)* | 0.89 (0.11)*** | 0.25 (0.12) | 0.62 (0.12)*** | 0.28 (0.12)* | 0.87 (0.14)*** |
| Bonus payment: Yes (Reference=No) | 0.16 (0.05)** | 0.15 (0.04)*** | 0.19 (0.04)*** | 0.12 (0.04)** | 0.18 (0.04)*** | 0.15 (0.04)*** | 0.29 (0.05)*** |
| Year 2011 (Reference=2010) |  |  | -0.14 (0.08) |  |  |  |  |
| Year 2012 (Reference=2010) |  |  | -0.24 (0.08)** |  |  |  |  |
| Year 2013 (Reference=2010) |  |  | -0.25 (0.08)** |  |  |  |  |
| Year 2014 (Reference=2010) |  |  | -0.17 (0.09) |  |  |  |  |
| Year 2015 (Reference=2010) |  |  | -0.19 (0.08)* |  |  |  |  |
| Year 2016 (Reference=2010) |  |  | -0.16 (0.1) |  |  |  |  |
| Constant | 0.54 (0.03)*** | 0.53 (0.03)*** | 0.84 (0.06)*** | 0.55 (0.03)*** | 0.64 (0.03)*** | 0.54 (0.03)*** | 0.54 (0.03)*** |
| Nurse-led follow-up: Yes (Reference=No) | 0.32 (0.03)*** | 0.3 (0.03)*** | 0.22 (0.04)*** | 0.2935543 (0.03)*** | 0.25 (0.03)*** | 0.31 (0.03)*** | 0.34 (0.03)*** |
| Constant | 0.44 (0.02)*** | 0.43 (0.02)*** | 0.64 (0.03)*** | 0.44 (0.02)*** | 0.58 (0.03)*** | 0.43 (0.02)*** | 0.46 (0.03)*** |
| Registration: Incentive (Reference=No incentive or obligation) | -0.18 (0.06)** | -0.16 (0.05)** | -0.18 (0.04)*** | -0.15 (0.05)** | -0.14 (0.06)** | -0.16 (0.05)** | -0.18 (0.05)** |
| Registration: Obligation (Reference=No incentive or obligation) | 0.08 (0.06) | 0.08 (0.06) | 0.06 (0.04) | 0.07 (0.05) | 0.06 (0.06) | 0.09 (0.06) | 0.20 (0.06)** |
| Year 2011 (Reference=2010) |  |  | -0.13 (0.06)* |  |  |  |  |
| Year 2012 (Reference=2010) |  |  | -0.22 (0.07)** |  |  |  |  |
| Year 2013 (Reference=2010) |  |  | -0.22 (0.07)** |  |  |  |  |
| Year 2014 (Reference=2010) |  |  | -0.14 (0.08) |  |  |  |  |
| Year 2015 (Reference=2010) |  |  | -0.17 (0.07)* |  |  |  |  |
| Year 2016 (Reference=2010) |  |  | -0.13 (0.1) |  |  |  |  |
| Constant | 0.62 (0.46)*** | 0.6 (0.43)*** | 0.92 (0.06)*** | 0.6 (0.04)*** | 0.73 (0.04)*** | 0.61 (0.04)*** | 0.63 (0.05)*** |
| Control access: Incentive (Reference=No incentive or obligation) | 0.24 (0.03)*** | 0.24 (0.03)*** | 0.1 (0.05)* | 0.23 (0.03)*** | 0.18 (0.05)*** | 0.23 (0.03)*** | 0.25 (0.03)*** |
| Control access: Obligation (Reference=No incentive or obligation) | 0.46 (0.02)*** | 0.44 (0.02)*** | 0.3 (0.03)*** | 0.43 (0.03)*** | 0.34 (0.04)*** | 0.45 (0.02)*** | 0.49 (0.02)*** |
| Year 2011 (Reference=2010) |  |  | -0.13 (0.06)* |  |  |  | 0.01 (0.03) |
| Year 2012 (Reference=2010) |  |  | -0.21 (0.06)** |  |  |  | 0.00 (0.03) |
| Year 2013 (Reference=2010) |  |  | -0.21 (0.07)** |  |  |  | 0.01 (0.03) |
| Year 2014 (Reference=2010) |  |  | -0.14 (0.07) |  |  |  | 0.05 (0.03) |
| Year 2015 (Reference=2010) |  |  | -0.15 (0.07)* |  |  |  | 0.08 (0.03)* |
| Year 2016 (Reference=2010) |  |  | -0.13 (0.08) |  |  |  | 0.1 (0.03)** |
| Constant | 0.27 (0.19)*** | 0.27 (0.19)*** | 0.68 (0.06)*** | 0.28 (0.02)*** | 0.46 (0.03)*** | 0.27 (0.02)*** | 0.24 (0.02)*** |
| Out of hours primary care: physicians rota: Yes (Reference=No) | -0.12 (0.05)* | -0.14 (0.04)** | -0.03 (0.05) | -0.13 (0.05)** | -0.07 (0.05) | -0.17 (0.05)*** | -0.25 (0.05)*** |
| Constant | 0.70 (0.04)*** | 0.7 (0.03)*** | 0.78 (0.04)*** | 0.69 (0.04)*** | 0.77 (0.04)*** | 0.74 (0.04)*** | 0.85 (0.05)*** |
| Registry: Yes (Reference=No) | -0.06 (0.05) | -0.05 (0.05) | -0.19 (0.04)*** | -0.05 (0.04) | -0.14 (0.04)** | -0.03 (0.05) | -0.04 (0.06) |
| Constant | 0.63 (0.04)*** | 0.61 (0.03)*** | 0.83 (0.03)*** | 0.61 (0.03)*** | 0.77 (0.03)*** | 0.61 (0.03)*** | 0.67 (0.04)*** |
| Guidelines: Yes (Reference=No) | 0.13 (0.06) | 0.12 (0.06)* | 0.02 (0.05) | 0.11 (0.06) | 0.06 (0.06) | 0.12 (0.06) | 0.14 (0.07) |
| Constant | 0.51 (0.06)*** | 0.50 (0.05)*** | 0.74 (0.05)*** | 0.51 (0.05)*** | 0.67 (0.05)*** | 0.51 (0.06)*** | 0.55 (0.06)*** |
| Task-shifting: Limited (Reference=No task-shifting)  Task-shifting: Extensive (Reference=No task-shifting)  Constant | 0.16 (0.05)**  0.38 (0.05)***  0.45 (0.03)*** | 0.16 (0.04)***  0.32 (0.04)***  0.45 (0.03)*** | 0.08 (0.05)  0.25 (0.05)***  0.66 (0.03)*** | 0.14 (0.05)**  0.32 (0.04)***  0.46 (0.03)*** | 0.10 (0.05)*  0.29 (0.05)***  0.60 (0.03)*** | 0.17 (0.04)***  0.35 (0.05)***  0.45 (0.03)*** | 0.25 (0.05)***  0.37 (0.05)***  0.45 (0.04)*** |

Notes: SE=Bootstrapped Standard Error (with 2,000 bootstrap replications). ***p<0.000, **p<0.01, *p<0.05. Year variables (time fixed effects) were included in models based on the results of log likelihood ratio tests – see Table S1.6 below.

**Table S6. Results of log likelihood ratio tests for inclusion of year variables (time fixed effects)**

|  | **Baseline model** | | **Baseline model with diabetes prevalence** | | **Baseline model with alcohol** | | **Baseline model with obesity** | | **Baseline model with smoking** | | **Baseline model with education** | | **Baseline model with income** | |
| --- | --- | --- | --- | --- | --- | --- | --- | --- | --- | --- | --- | --- | --- | --- |
| **Variable** | **LR chi2 (6df)** | **Prob>chi2** | **LR chi2 (6df)** | **Prob>chi2** | **LR chi2 (6df)** | **Prob>chi2** | **LR chi2 (6df)** | **Prob>chi2** | **LR chi2 (6df)** | **Prob>chi2** | **LR chi2 (6df)** | **Prob>chi2** | **LR chi2 (6df)** | **Prob>chi2** |
| Curative care beds, per 1,000 population | 2.59 | 0.858 | 3.66 | 0.722 | 10.79 | 0.095 | 2.45 | 0.874 | 3.61 | 0.730 | 1.84 | 0.934 | 5.93 | 0.431 |
| Availability and use of Electronic Health Records (EHR) by GPs | 2.59 | 0.858 | 3.50 | 0.744 | 10.8 | 0.095 | 2.41 | 0.879 | 3.20 | 0.783 | 2.20 | 0.901 | 4.43 | 0.619 |
| Bonus payment | 1.88 | 0.930 | 2.41 | 0.878 | 13.86 | 0.031 | 2.11 | 0.909 | 4.71 | 0.582 | 1.30 | 0.972 | 3.54 | 0.739 |
| Nurse-led follow-up | 1.35 | 0.969 | 1.86 | 0.932 | 11.99 | 0.062 | 1.48 | 0.961 | 4.35 | 0.629 | 0.49 | 0.998 | 4.29 | 0.637 |
| Registration | 2.18 | 0.903 | 2.94 | 0.816 | 13.18 | 0.040 | 2.19 | 0.902 | 4.12 | 0.660 | 1.54 | 0.957 | 5.43 | 0.490 |
| Control access | 4.71 | 0.581 | 6.93 | 0.327 | 17.78 | 0.007 | 4.97 | 0.548 | 5.36 | 0.499 | 3.91 | 0.689 | 16.88 | 0.010 |
| Out of hours primary care: physicians rota | 1.88 | 0.930 | 2.52 | 0.867 | 10.32 | 0.112 | 1.93 | 0.926 | 3.46 | 0.750 | 1.27 | 0.973 | 4.13 | 0.659 |
| Registry | 2.25 | 0.896 | 3.16 | 0.788 | 12.41 | 0.053 | 2.25 | 0.896 | 3.56 | 0.736 | 1.80 | 0.937 | 5.19 | 0.520 |
| Guidelines | 1.94 | 0.925 | 2.69 | 0.846 | 10.41 | 0.108 | 2.06 | 0.914 | 3.32 | 0.768 | 1.54 | 0.957 | 4.63 | 0.592 |
| Task-shifting | 1.82 | 0.935 | 2.60 | 0.857 | 12.17 | 0.058 | 2.13 | 0.907 | 3.62 | 0.728 | 1.44 | 0.963 | 5.08 | 0.533 |

**Figure S1. Generalist medical practitioners, per 1,000 population, average 2010-2016**

**Figure S2. Diabetes hospital admissions per 100,000 population, average 2010-2016**

**Figure S3.** **Admission based diabetes lower extremity amputation, per 100,000 population, average 2010-2016**

**Figure S4. Diabetes prevalence, percentage of population (age-standardised) , average 2010-2016**

**Figure S5. Alcohol consumption, litres per capita, average 2010-2016**

**Figure S6. Prevalence of obesity among adults aged 18 years and over, (%), average 2010-2016**

**Figure S7. Smoking, percentage of population aged 15+, average 2010-2016**

**Figure S8. Severe material deprivation, percentage of population, average 2010-2016**

**Figure S9. Upper secondary level education, % of population aged 25-64 years, average 2010-2016**

**Figure S10. Income per capita, US$ Purchasing Power Parity, average 2010-2016**

**Figure S11. Percentage of total unemployed population unemployed for one year or more, average 2010-2016**

**Figure S12. Curative care beds, per 1,000 population, average 2010-2016**

**Figure S13. Availability and use of Electronic Health Records by GPs, average 2010-2016**
